# Lysosomal Cathepsin Release Is Required for NLRP3-Inflammasome Activation by *Mycobacterium tuberculosis* in Infected Macrophages

**DOI:** 10.3389/fimmu.2018.01427

**Published:** 2018-06-21

**Authors:** Eduardo P. Amaral, Nicolas Riteau, Mahtab Moayeri, Nolan Maier, Katrin D. Mayer-Barber, Rosana M. Pereira, Silvia L. Lage, Andre Kubler, William R. Bishai, Maria R. D’Império-Lima, Alan Sher, Bruno B. Andrade

**Affiliations:** ^1^Immunobiology Section, Laboratory of Parasitic Diseases, National Institute of Allergy and Infectious Diseases, National Institutes of Health, Bethesda, MD, United States; ^2^Inflammation and Innate Immunity Unit, Laboratory of Clinical Immunology and Microbiology, National Institute of Allergy and Infectious Diseases, National Institutes of Health, Bethesda, MD, United States; ^3^Laboratory of Immunology of Infectious Diseases, Department of Immunology, Institute of Biomedical Science, University of São Paulo, São Paulo, Brazil; ^4^Clinical and Molecular Retrovirology Section, Laboratory of Immunoregulation, National Institute of Allergy and Infectious Diseases, National Institutes of Health, Bethesda, MD, United States; ^5^Department of Medicine, Imperial College London, London, United Kingdom; ^6^Center for Tuberculosis Research, Johns Hopkins University School of Medicine, Baltimore, MD, United States; ^7^Instituto Gonçalo Moniz, Fundação Oswaldo Cruz, Salvador, Bahia, Brazil; ^8^Multinational Organization Network Sponsoring Translational and Epidemiological Research (MONSTER) Initiative, José Silveira Foundation, Salvador, Brazil; ^9^Wellcome Centre for Infectious Disease Research in Africa, Institute of Infectious Disease and Molecular Medicine, University of Cape Town, Cape Town, South Africa; ^10^Division of Infectious Diseases, Department of Medicine, Vanderbilt University School of Medicine, Nashville, TN, United States; ^11^Universidade Salvador (UNIFACS), Laureate University, Salvador, Bahia, Brazil; ^12^Escola Bahiana de Medicina e Saúde Pública, Salvador, Bahia, Brazil

**Keywords:** cathepsin B, tuberculosis, IL-1β, inflammasome, ESAT-6 secretion system

## Abstract

Lysosomal cathepsin B (CTSB) has been proposed to play a role in the induction of acute inflammation. We hypothesised that the presence of active CTSB in the cytosol is crucial for NLRP3-inflammasome assembly and, consequently, for mature IL-1β generation after mycobacterial infection *in vitro*. Elevated levels of CTSB was observed in the lungs of mice and rabbits following infection with *Mycobacterium tuberculosis* (Mtb) H37Rv as well as in plasma from acute tuberculosis patients. H37Rv-infected murine bone marrow-derived macrophages (BMDMs) displayed both lysosomal leakage, with release of CTSB into the cytosol, as well as increased levels of mature IL-1β. These responses were diminished in BMDM infected with a mutant H37Rv deficient in ESAT-6 expression. Pharmacological inhibition of cathepsin activity with CA074-Me resulted in a substantial reduction of both mature IL-1β production and caspase-1 activation in infected macrophages. Moreover, cathepsin inhibition abolished the interaction between NLRP3 and ASC, measured by immunofluorescence imaging in H37Rv-infected macrophages, demonstrating a critical role of the enzyme in NLRP3-inflammasome activation. These observations suggest that during Mtb infection, lysosomal release of activated CTSB and possibly other cathepsins inhibitable by CA07-Me is critical for the induction of inflammasome-mediated IL-1β processing by regulating NLRP3-inflammasome assembly in the cytosol.

## Introduction

The pro-inflammatory cytokine IL-1β is thought to play a major role in host protection against *Mycobacterium tuberculosis* (Mtb) infection (1–8). Thus, mice genetically deficient in IL-1β, IL-1α, or IL-1 receptor signaling are extremely susceptible to Mtb infection (1, 2, 4, 7) while *in vitro* stimulation of Mtb-infected macrophages with IL-1β results in reduced mycobacterial burdens (7). Several explanations for the anti-mycobacterial activity of IL-1β have been proposed. For example, IL-1β has been shown to facilitate phagolysosomal fusion, which enhances the mycobactericidal activity of host macrophages (9). In addition, IL-1β through its induction of prostaglandin E2 could influence the cell death modality of Mtb-infected macrophages favoring an apoptotic over necrotic fate thus containing bacterial spread (7, 10–13). This could occur in part thru the generation of cyclooxygenase-2, which plays critical role in plasma membrane repair (7, 10).

While there is strong evidence demonstrating a beneficial role of IL-1β in Mtb infection *in vitro* and *in vivo* (2, 4, 7, 9), excessive production of this cytokine has been associated with more severe tuberculosis (TB) disease and increased lung damage (14–16). Thus, understanding the nuances of IL-1β function in TB is important in the design of therapeutic interventions that act by altering the levels and activity of this major pro-inflammatory cytokine.

*In vitro*, Mtb infection of macrophages has been shown to induce inflammatory responses *via* activation of the NLRP3-inflammasome, leading to secretion of mature IL-1β (5, 17). Additional studies have demonstrated that Mtb can elicit NLRP3-inflammasome through antigens encoded by the mycobacterial genome known as region of difference 1 (RD-1) (5, 17, 18). Among the Mtb proteins included in RD-1 encoded group, the early-secreted antigen (ESAT-6) has been identified as the major mycobacterial product responsible for IL-1β induction and does so through an NLRP3-dependent pathway (5). ESAT-6 is secreted from Mtb through a specialized protein secretion system of the bacterium called ESX-1 (19–21) that has been proposed to mediate pore formation in the phagosomal membranes thus facillitating mycobacterial escape from that organelle (22, 23). Several stimuli elicit NLRP3 activation leading to the recruitment of the adaptor protein ASC (apoptosis-associated speck-like protein containing a carboxy-terminal CARD), which is required for caspase-1 recruitment and activation, culminating in the NLRP3-inflammasome complex formation (5, 24). Once assembled, the NLRP3-inflammasome complex enables the maturation of pro-IL1β and release of its mature form in a caspase-1-dependent manner. However, the exact mechanism of NLRP3-inflammasome activation by mycobacteria is poorly understood.

Several studies have described the involvement of lysosomal cathepsins in NLRP3-dependent IL-1β production (25–28). Cathepsins are a class of proteolytic enzymes which display diversity in terms of their structural and/or functional features. The lysosomal cathepsin family can be divided in three subsets, the aspartic cathepsins (D and E), serine cathepsins (A and G), and cysteine cathepsins (B, C, F, H, K, L, O, S, V, X, and W). Cathepsin B (CTSB) expression is ubiquitous, and usually expressed in higher levels than other cathepsins in some tissues (29, 30). Of note, CTSB can be distinguished from cathepsin family members on the basis of its activity as either an endo- or exopeptidases at different pH ranges (30). Inside cells, CTSB regulates several functions, including cytokine exocytosis, protein cleavage inside the lysosome, and the induction of cell death (5, 17, 31–37). Interestingly, CTSB activation either inside the lysosome or in the cytosol has been proposed to promote NLRP3-inflammasome activation and IL-1β production in experimental settings (38–40). Furthermore, CTSB has been described in some settings to directly cleave caspase-1 and caspase-11 which could further promote IL-1β maturation (30–33, 41). Nonetheless, in many experimental models it has been difficult to demonstrate a direct role for CTSB cleavage in IL-1β maturation (30, 36, 42) suggesting that its role may be upstream of inflammasome activation.

Since ESAT-6 has been associated with both membrane pore formation and IL-1β production by Mtb, we asked in the present study whether the ESAT-6 induced release of lysosomal CTSB might link these two phenomena. Using macrophages infected *in vitro* with Mtb, we demonstrate that when secreted into the cytosol by an ESAT-6-dependent mechanism, lysosomal cathepsin activity inhibitable by CA074-Me is crucial for NLRP3-inflammasome activation and consequently, for mature IL-1β generation. Previous studies have shown that IL-1β control of Mtb infection is inflammasome independent. Thus, the cathepsin-dependent pathway for IL-1β generation we describe could serve as a potential target for reducing Mtb induced inflammation without compromising host resistance to the pathogen.

## Results

### Mtb Infection Triggers Increased CTSB Levels and Activity

**Figure 1:**
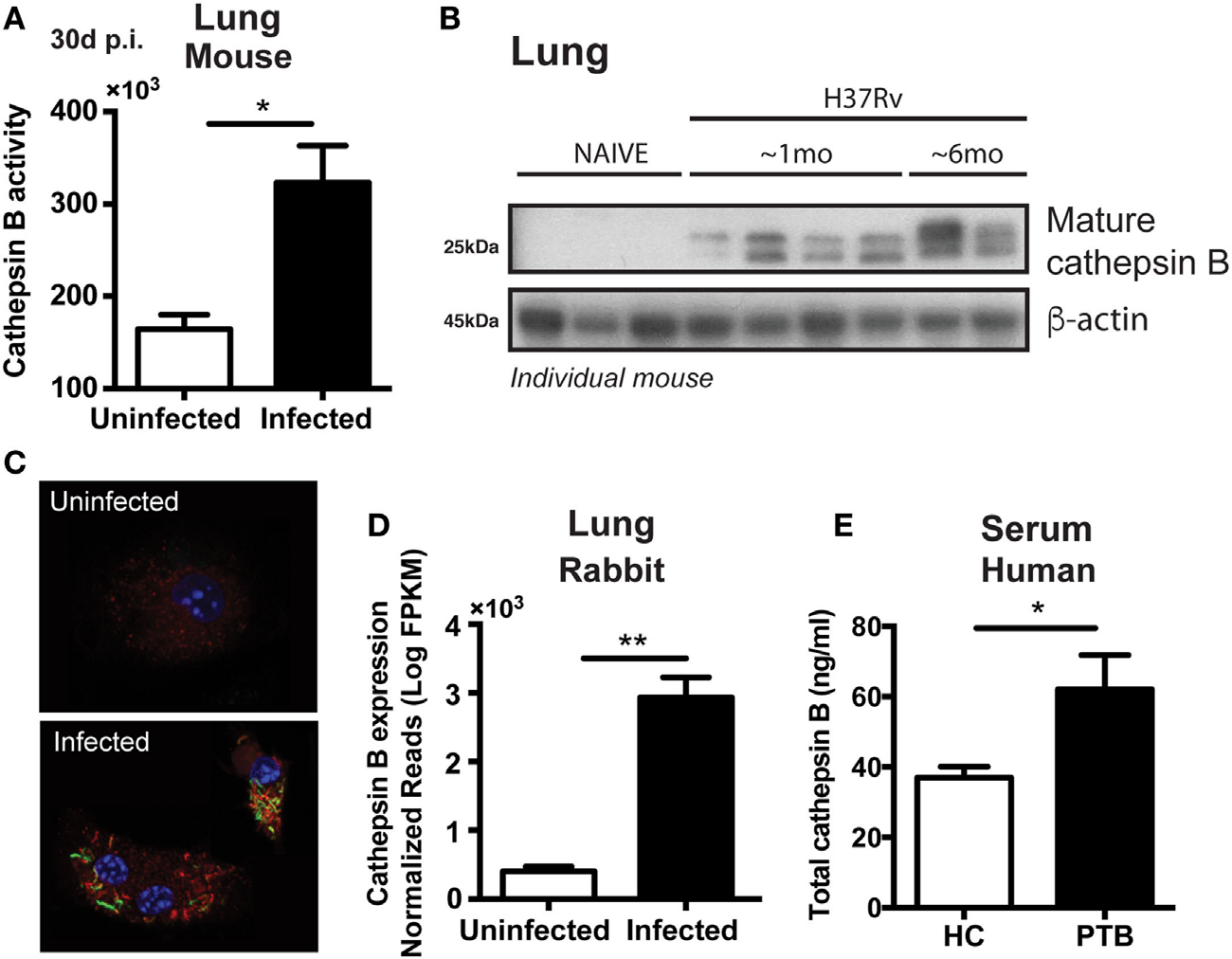
*Mycobacterium tuberculosis* (Mtb) infection upregulates both cathepsin B (CTSB) expression and enzymatic activity. **(A)** CTSB activity and **(B)** expression of mature CTSB were measured in the lungs of H37Rv-infected mice after 1 month or 6 months post-infection (mean ± SEM, *n* = 5). **(C)** CTSB activity (red) in macrophages infected (multiplicity of infection: 3) with GFP-H37Rv Mtb strain (green) was analyzed by confocal microscopy (magnification: 600×). DAPI (blue) was used for nuclear staining. Representative images are shown. **(D)** CTSB messenger RNA expression was quantified in lung granulomatous lesions from rabbits infected with Mtb H37Rv at day 42 post-infection (mean ± SEM, *n* = 3–4). **(E)** Total CTSB levels in plasma from patients with active pulmonary TB (PTB; mean ± SEM, *n* = 13) and health controls (HC; mean ± SEM, *n* = 12). Data were analyzed using the Mann–Whitney *U* test (*p < 0.05; **p < 0.01). Data are representative of at least two separate experiments performed.

We first investigated whether Mtb infection increases the expression or activity of CTSB in macrophages. To do so, we quantified levels of the protease in lung tissues in two different animal models of pulmonary TB (mice and rabbits), in plasma from patients with active TB as well as in Mtb-infected bone marrow-derived macrophages (BMDMs). CTSB enzyme activity was found increased by two-fold in the lungs from mice infected with Mtb on day 30 post-infection (Figure 1A) and was further elevated after 6 months of infection (Figure 1B). Furthermore, *in situ* analysis of CTSB activity was evaluated during BMDM infection and was enhanced in the presence of Mtb (Figure 1C). In the latter assays, enzyme activity was detected in close association with the bacteria in lysosomal-like structures. These findings were supported by data showing increased CTSB gene expression in the lungs of Mtb-infected rabbits (Figure 1D) and elevated protein levels in plasma from patients with active TB (Figure 1E). Together these results demonstrated that Mtb increases CTSB expression in different host species and that this response is detectable both *in vivo* and *in vitro*. In the case of the *in vivo* findings, the observed elevation in CTSB levels could reflect increased cellular exocytosis and cell damage in addition to the induction of enzyme synthesis.

### Mature CTSB Is Released From Lysosomes Into the Cytosol by an ESAT-6-Dependent Mechanism

**Figure 2:**
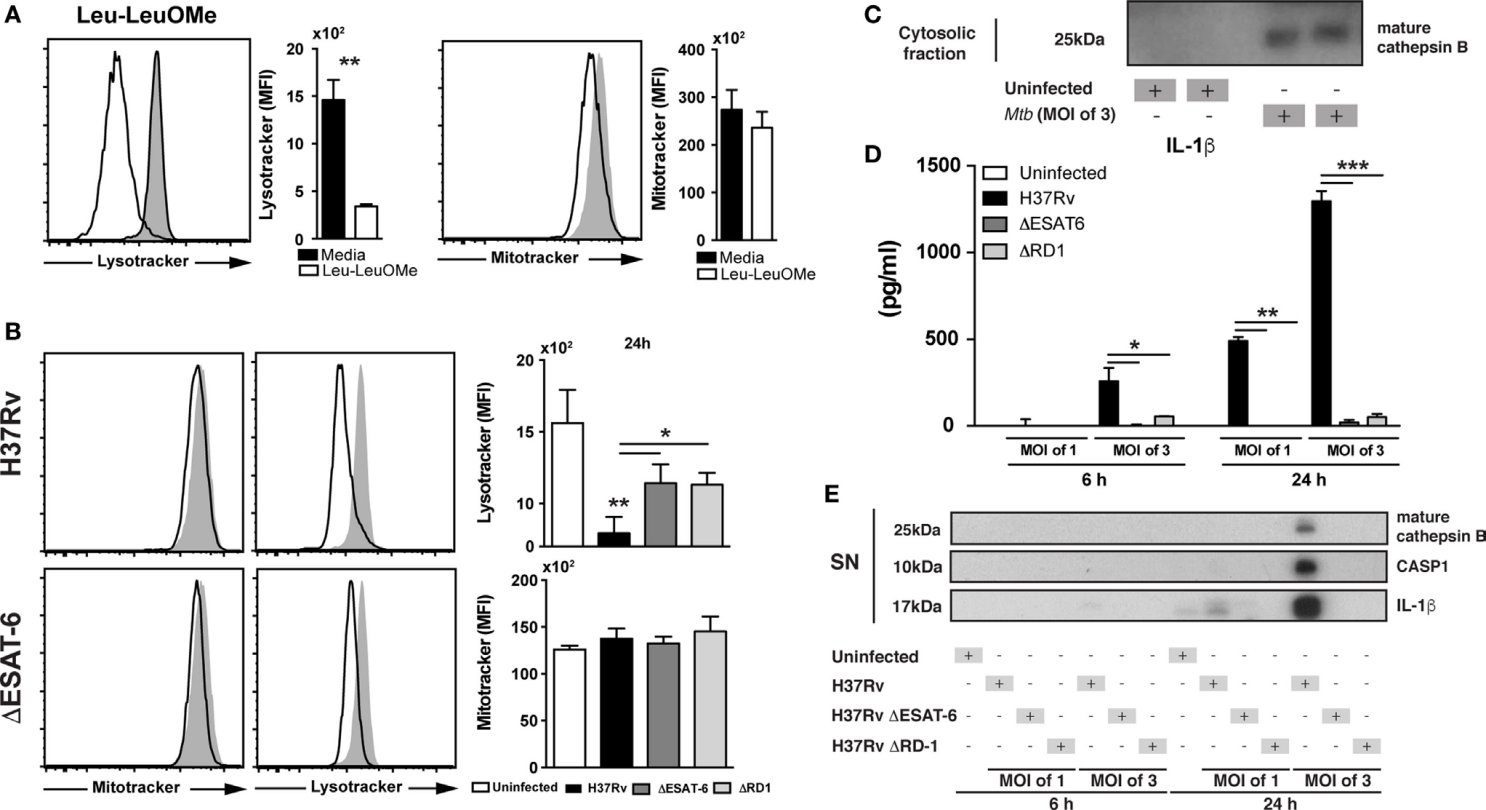
*Mycobacterium tuberculosis* (Mtb) infection induces lysosomal leakage dependent on ESAT6 and leads to release of mature cathepsin B (CTSB) and IL-1β production. Cells were stained with lysotracker and mitotracker to verify lysosomal leakage or outer mitochondrial membrane potential disturbance at 24 h p.i. with Mtb H37Rv strain (MOI of 3), respectively. **(A)** Cells were stimulated with Leu-o-Leu as a positive control of lysosomal leakage. **(B)** Bone marrow-derived macrophages (BMDMs) were infected with H37Rv, ΔESAT6 H37Rv, or ΔRD1 H37Rv Mtb strains at MOI of 3 as described in Section “Materials and Methods.” Mtb H37Rv induced lysosomal leakage dependent of ESAT6. **(C)** Cytosolic fraction from BMDMs infected with Mtb H37Rv, showing the presence of mature CTSB. **(D,E)** BMDMs were infected with H37Rv, ΔESAT6 H37Rv, or ΔRD1 H37Rv strains at MOI of 1 or MOI of 3 as described in Section “Materials and Methods.” **(D)** IL-1β production was measured by ELISA and **(E)** expression of mature IL-1β, activated caspases-1, and mature CTSB in the supernatant of cells was assessed by western blotting. Differences were analyzed using the Mann–Whitney *U* test (between two groups) or the Kruskal–Wallis test with Dunn’s multiple comparisons *ad hoc* test. Significant differences were observed for the indicated experimental conditions (*p < 0.05; **p < 0.01). Bars and line represent mean and SEM, respectively. Data are representative of three independent experiments using triplicate biological samples.

We next asked whether during Mtb infection of macrophages, CTSB was released from the lysosomes into the cytosol. Lysosomal destabilization was analyzed by staining BMDM with the LysoTracker dye, and fluorescence intensity of the dye measured by flow cytometry (43, 44). As a positive control for lysosomal integrity loss, we stimulated cells with Leu-leu-OMe (a known inducer of lysosomal destabilization) (43) and then checked lysosomal leakage by using LysoTracker and loss of mitochondrial membrane potential by using Mitotracker. Interestingly, previous studies have also shown that Leu-leu-OMe-induced lysosomal leakage facilitate the release of CTSB into the cytosol (43, 45, 46), supporting the use of Leu-leu-OMe as a gold-standard positive control for lysosomal destabilization. As expected, Leu-leu-OMe treated cells displayed reduced fluorescent intensity for Lysotracker dye but not for Mitotracker (Figure 2A). Macrophages infected with Mtb showed reduced staining with Lysotracker comparable to that triggered by Leu-leu-OMe (Figures 2A,B) and was not accompanied by changes in mitochondrial permeability (Figure 2B). Importantly, Mtb strains deficient in ESAT-6 or RD-1 triggered significant smaller reductions in Lysotracker staining than the wild-type (WT) H37Rv parental strain. Together the above findings indicated that Mtb induces lysosomal destabilization by a mechanism partially dependent on ESAT-6/RD-1.

We next investigated whether the lysosomal disruption induced by Mtb results in the release of CTSB into the cytosol. We adapted a previously described method using saponin to discriminate lysosomal versus cytosolic content and measured the presence of CTSB in the cytosol at different doses (43) (Figure S1 in Supplementary Material). We found that Mtb-infected BMDM displayed increased levels of mature CTSB inside the cytosol as compared to uninfected controls. These data confirmed that lysosomal leakage induced by Mtb is indeed associated with release of mature CTSB into the cytosol (Figure 2C).

*Mycobacterium tuberculosis* ESAT-6 is a highly immunogenic mycobacterial antigen, which has been described to induce pore formation in the phagosome membrane thus facilitating bacterial escape into the cytosol (23). Moreover, it has been reported that ESAT-6 is required for NLRP3-inflammasome activation and the subsequent cleavage of pro-IL-1β into its mature form (5). We also observed reduced levels of mature IL-1β in supernatants from BMDM cultured with ΔESAT6 H37Rv or ΔRD1 H37Rv strains compared to those infected with WT H37Rv (Figure 2D). In addition, we measured mature CTSB, cleaved caspase-1, and mature IL-1β in supernatants from Mtb-infected macrophages by western blotting (WB). Following H37Rv infection, we found increased levels of mature CTSB, cleaved caspase-1, and mature IL-1β from infected macrophages, the latter observation confirming that the changes in IL-1β levels measured by ELISA (Figure 2D) reflect alterations in the mature cytokine. These products were not detected in supernatants from cells infected with ΔESAT6 H37Rv or ΔRD1 H37Rv strains (Figure 2E). Taken together, the above findings suggested that ESAT-6 facilitates the secretion of the activated CTSB into the cytosol which is associated with enhanced IL-1β maturation.

### Inhibition of Cathepsin Activity by CA074-Me Blocks IL-1β Production Induced by Either Mtb Infection or Cytosolic ESAT-6

**Figure 3:**
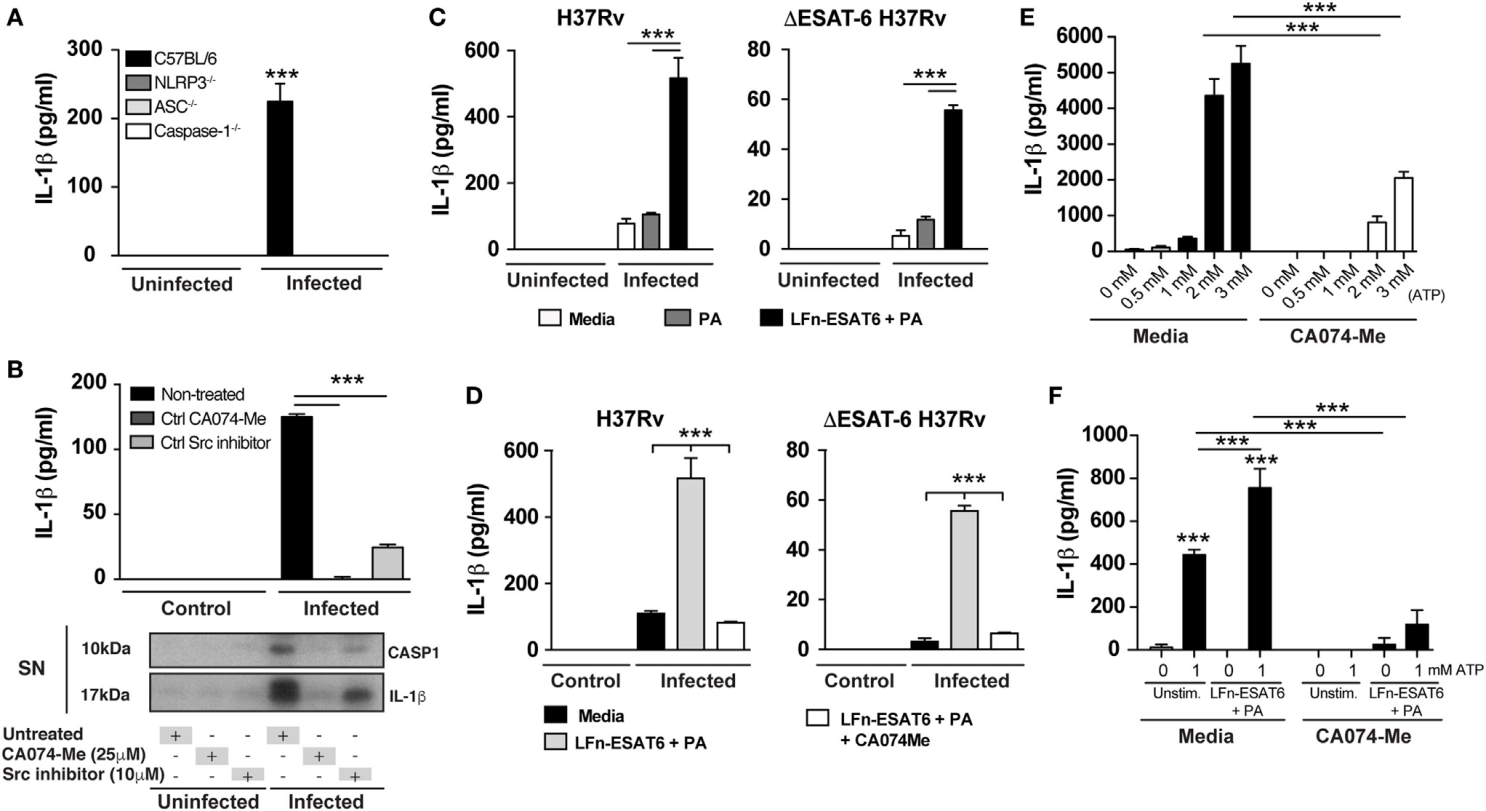
*Mycobacterium tuberculosis* (Mtb) ESAT-6-induced IL-1β production is diminished by cathepsin B inhibition. Macrophages were infected with Mtb H37Rv or ΔESAT6 strains at MOI of 3 as described in Section “Materials and Methods.” **(A,B)** IL-1β production was measured in the supernatants collected from Mtb-infected deficient macrophages or following different treatments as indicated. **(B)** Mature IL-1β and cleaved caspase-1 were analyzed by western blotting. **(C,D)** Infected macrophages were stimulated with PA (20 µg/mL) or LFn-ESAT6/PA (20 µg/mL) and IL-1β production was quantified by ELISA after 24 h of infection. **(E,F)** Bone marrow-derived macrophages were primed with LPS (0.2 µg/mL) for 1 h and further stimulated or not with LFn-ESAT/PA (10 µg/mL each) for 3 h. Furthermore, cells were stimulated with eATP (1 mM) for 25 min and IL-1β production was measured in culture supernatants. Statistical differences observed are shown for each indicated groups (***p < 0.001). The data represent the mean ± SEM of samples in triplicate. Data are from at least three independent experiments.

To confirm the requirement of NLRP3-inflammasome complex for IL-1β production in our experimental system, we infected BMDM from NLRP3^−/−^, ASC^−/−^, caspase-1^−/−^, and WT mice with Mtb H37Rv and measured IL-1β in supernatants from macrophages 24 h later. As expected, IL-1β production induced by Mtb was completely abolished in the supernatants from macrophages deficient for NLRP3, ASC, and caspase-1 (Figure 3A).

We next investigated whether lysosomal cathepsin activity is required for release of mature IL-1β. Macrophages were infected with H37Rv in the presence or absence of CA074-Me, a potent inhibitor of CTSB activity and in certain conditions other cathepsins (36, 43), and mature IL-1β as well as cleaved caspase-1 were measured in supernatants at 6 and 24 h after infection. We found that the release of both cleaved caspase-1 and mature IL-1β secretion was completely inhibited after CA074-Me treatment (Figure 3B). Of note, CA074-Me treatment does not affect pro-IL-1β expression as well as cell death induction as previously reported (Figures S2A,C in Supplementary Material) (17, 43, 47). Interestingly, inhibition of the tyrosine kinase Src also blocked IL-1β and cleaved caspase-1 secretion. This effect correlated with a reduction in lysosomal leakage following Src inhibitor treatment (Figure S3 in Supplementary Material).

The above results taken together demonstrated that Mtb ESAT-6 expression induces lysosomal perturbation, mature CTSB release into cytosol, and mature IL-1β secretion after Mtb infection. It has been previously established that ESAT-6 facilitates the escape of Mtb into the cytosol from phagolysosomes (23). Thus, it is challenging to determine whether CTSB release into the cytosol following lysosomal leakage occurs due to ESAT-6-induced pore formation on phagolysosomal membranes or directly through ESAT-6 action in the cytosol. To distinguish these two mechanisms, we performed experiments using a previously described system involving an anthrax toxin LFn-fusion protein to deliver recombinant ESAT-6 into the cytosol (48–51). In these experiments, we infected BMDM with H37Rv and ΔESAT6 H37Rv strains in the presence or not of the LFn-ESAT-6/PA complex (50, 51) and then measured IL-1β production 24 h post-infection. Delivery of ESAT-6 into the cytosol enhanced IL-1β production by H37Rv-infected macrophages and importantly restored IL-1β production in ΔESAT6 H37Rv-infected cells (Figure 3C). Moreover, the IL-1β response induced by ESAT-6 delivery into the cytosol could be abolished by treatment of the cells with the CA074-Me, a potent CTSB inhibitor (Figure 3D).

Although the expression of recombinant ESAT-6 (rESAT-6) in the cytosol resulted in IL-1β production, this effect was nonetheless dependent on Mtb infection (Figure 3C). To investigate whether rESAT-6 can enhance IL-1β production induced by other known NLRP3-inflammasome stimuli in a non-infectious context, we used the well-known model of NLRP3-inflammasome activation through extracellular ATP stimulation. We first performed a dose titration of eATP concentration to determine non-saturating levels of IL-1β production without inducing cell death *via* P2X7 receptor (Figure 3E) and determined the optimal concentration at 1 mM. We then primed macrophages with LPS (0.2 µg/mL) for 1 h (as first signal for pro-IL-1β expression), and then rESAT-6 was delivered into the cytosol by stimulating cells with LFn-ESAT6/PA (10 µg/mL) for an additional 3 h. Finally, macrophages were incubated with eATP (1 mM) for 25 min and IL-1β production measured by ELISA. We found that cytosolic delivery of ESAT-6 amplified IL-1β production induced by ATP in a CTSB-dependent manner (Figure 3F). Thus, these results suggested that the presence of ESAT-6 inside the cytosol can drive NLRP3-inflammasome activation independent of Mtb infection and that CTSB activity is required for this activation.

### CA074-Me Blocks ASC Speck Formation in Mtb-Infected Macrophages

**Figure 4:**
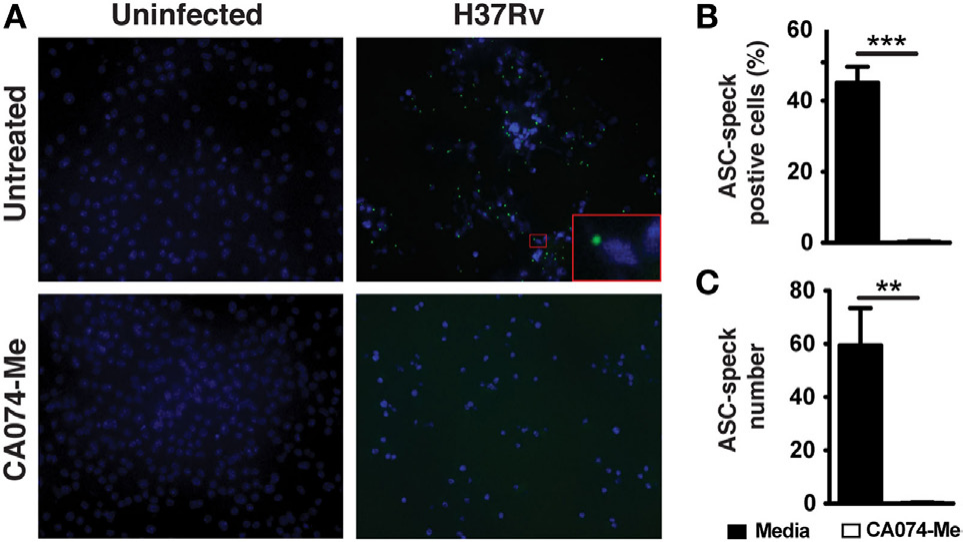
Cathepsin B (CTSB) activity mediates ASC speck formation. Macrophages were infected with *Mycobacterium tuberculosis* H37Rv MOI of 3 as described in Section “Materials and Methods” and cells were treated or not with the CTSB inhibitor CA074-Me as indicated. **(A)** ASC speck formation in infected macrophages treated or not with CA074-Me (25 µM) was analyzed by fluorescence microscopy. **(B)** The frequency of cells ASC speck positives **(C)** and number of ASC speck were quantified by using the Image J software. Differences were analyzed using the Mann–Whitney *U* test. Statistical differences observed are shown for each indicated groups (***p < 0.001). Data represent mean ± SEM of samples ran in triplicate. Results are representative at least two independent experiments.

We next addressed the mechanism by which cytosolic CTSB might promote IL-1β production. ASC speck formation is an ultrastructural hallmark of NLRP3-inflammasome activation (52). These structures are formed by the accumulation of ASC and cleaved caspase-1 bound to an NLRP3 platform, thus promoting the generation of mature IL-1β (52). We wondered whether lysosomal cathepsin activity is essential for ASC speck formation as a result of NLRP3-inflammasome assembly. To test this hypothesis, we infected macrophages with Mtb H37Rv and cells were incubated in the presence or absence of the CA074-Me cathepsin inhibitor. After 24 h of infection, speck of ASC was stained using specific antibodies and samples analyzed by fluorescence microscope. Numerous ASC specks were detected in Mtb-infected macrophages (Figures 4A–C). However, these structures were barely detectable Mtb-infected macrophages treated with CA074-Me (Figures 4A–C). Thus, these data indicated that the suppression of mature IL-1β production induced by lysosomal cathepsin inhibitor CA074-Me (Figures 2D,E) is associated with defective NLRP3-inflammasome assembly and consequently ASC speck formation following Mtb infection.

## Discussion

Studies in murine models have identified IL-1β production as a critical factor in host resistance to Mtb (1, 2, 7, 9), but in certain settings the cytokine can also contribute to immunopathology (14, 16). It is well established that Mtb triggers IL-1β production in infected macrophages as a consequence of NLRP3-inflammasome activation *in vitro* (5, 17, 53, 54). Nevertheless, the specific mechanism by which NLRP3-inflammasome is activated during Mtb infection is poorly understood. The findings reported here reveal a previously unappreciated role of ESAT-6-dependent cathepsin activity in NLRP3-inflammasome assembly and IL-1β production in Mtb-infected macrophages.

The immunodominant ESAT-6 virulence factor plays major roles in the Mtb host interaction. ESAT-6-deficient mycobacteria are unable to induce necrotic macrophage death leading to low degree of bacterial spread to the extracellular *milieu* (17, 23, 47). In addition, ESAT-6 has been shown to be required for bacterial escape from the phagosome into the cytosol which promotes more rapid induction of necrosis *in vitro* and *in vivo* (23, 55). As would be expected from its function in necrosis, mice infected with ESAT-6-deficient Mtb display mild disease compared with WT Mtb-infected animals (18, 19, 56). This avirulent phenotype is associated with reduced levels of inflammation, oxidative stress, and cytokine signaling (12, 50, 51). ESAT-6 is also known to trigger mature IL-1β production, although the mechanism involved has been unclear. As shown in the present study, ESAT-6-dependent IL-1β production was completely abolished following treatment of macrophage cultures with a CTSB inhibitor, CA074-Me. In addition, we found that delivery of ESAT-6 into cytosol restores IL-1β production during macrophage infection with ΔESAT-6-Mtb and in uninfected macrophages enhances IL-1β production mediated by eATP *via* P2X7 activation. In both situations, the effects of ESAT-6 on IL-1β production were blocked by treatment with CA074-Me. Unexpectedly, genetic deficiency in CTSB or cathepsin L (CTSL) actually resulted in enhanced IL-1β (Figure S3 in Supplementary Material). Similar findings have been reported in CTSB-deficient or CTSL-deficient macrophages stimulated with uric acid or alum, and could result from the overexpression in the relevant deficient mouse strains of lysosomal enzymes with redundant function (30, 57). The use of CA074-Me for inhibiting CTSB is appropriate and justified because of its properties of high plasma membrane permeability and quick conversion to its non-methylated form, CA074, that has been shown to interfere specifically with CTSB activity (58–64). Importantly, CA074-Me does not affect other enzymes such as caspase-1, known to be essential for pro-IL-1β cleavage (36, 65). Finally, CTSB is found at relatively high levels and is widely distributed in the lysosomes of cells in various tissues (30), making it a prominent target for CA074-Me inhibition at the low drug concentration used here (64). While, we cannot at this stage formally rule out the participation of other related proteases, our data strongly support a major role for CTSB activation in Mtb induced IL-1β maturation.

Our data also argue that the release of mature CTSB in the cytosol is a critical step for NLRP3-inflammasome activation by facilitating interaction between NLRP3 and ASC, culminating in the formation of ASC specks. While CTSB inhibition clearly blocked Mtb-induced inflammasome assembly, we also observed inhibition of NLRP3 and ASC interaction in Mtb-infected macrophages following treatment with Src inhibitor (Figure S2C in Supplementary Material). Indeed, Src activity has been reported to be involved in mediating the secretion of proteins such as CTSB and CTSD, and its inhibition also abolishes IL-1β production in macrophage cultures stimulated with LPS and monosodium urate crystals (66). In fact, we observed that Src inhibition prevents mature CTSB release into the cytosol in Mtb-infected macrophages (Figure S2D in Supplementary Material).

Direct physical interaction between recombinant NLRP3 and CTSB proteins has been previously reported (27), supporting our hypothesis that CTSB can take part in NLRP3-inflammasome complex assembly as well as ASC speck formation. Whether the molecular mechanism by which CTSB promotes NLRP3-ASC complex formation by interfering the regulation of other proteins implied in the assembly of this complex such as NEK7/vimentin (67) remains to be determined.

The generation of ASC speck has been associated with high levels of IL-1β production and promotion of inflammation (52). It is known that excessive production of IL-1β cytokines is detrimental for host, since it is associated with intense inflammation and increased immunopathology. Mishra and colleagues have shown that detrimental neutrophilic inflammation in severe forms of TB is associated with elevated IL-1β production, which can be repressed by nitric oxide (16). In addition, nitric oxide inhibits IL-1β-mediated inflammation by repressing the caspase-1-dependent cleavage of pro-IL-1β (14). More recently, inflammasome signaling has been described as process in TB-associated immune reconstitution inflammatory syndrome, highlighting the importance of the inflammasome in clinical settings related to TB disease (68–74). In experimental models *in vivo*, mice deficient in IL-1β or IL-1R are extremely susceptible to Mtb infection, whereas NLRP3, ASC, and caspase-1 deficient mice do not display this phenotype (4, 53, 54). Interestingly, ASC has also been implicated granuloma maintenance during chronic phase of Mtb infection (53). These findings indicate that another inflammasome-independent mechanism leading to post-translational processing of pro-IL-1β may operate *in vivo*. One possibility is that enzymes, such as metalloproteinases and/or cathepsins, secreted during inflammation or released by cells undergoing cytolysis/necrosis may be responsible for the cleavage of pro-IL-1β in the extracellular *milieu* as described in other experimental models (75–77). Interestingly, in an animal model of viral myocarditis mice deficient in CTSB showed improved survival, reduced inflammatory cell tissue infiltration, and reduced IL-1β production, suggesting the CTSB aggravates the disease through activating the inflammasome-dependent IL-1β production and promoting pyroptosis (78).

**Figure 5:**
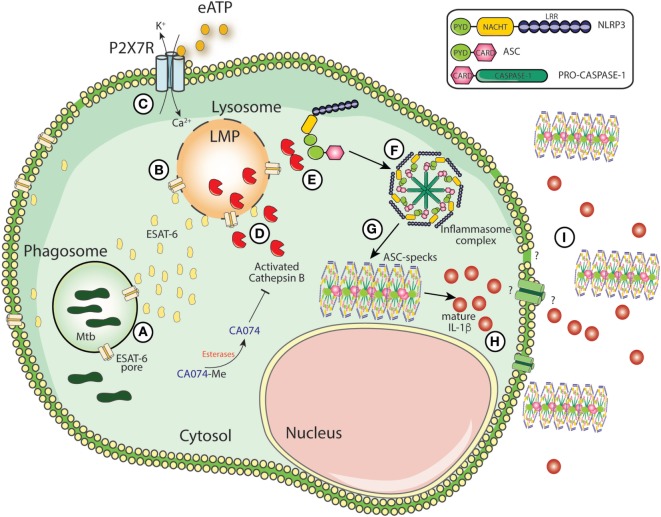
Schematic model for mature IL-1β generation during *Mycobacterium tuberculosis* (Mtb) infection. The secretion of ESAT-6 in phagosomes by Mtb induces pore formation on phagosomal membranes, facilitating the leakage of ESAT-6 into cytosol and bacterial escape to cytosolic compartment **(A)**. Once in the cytosol, ESAT-6 may induce pore formation on cellular membranes which could contribute to lysosomal perturbation (LMP) **(B)**. Simultaneously, extracellular ATP is recognized by P2X7 receptor and amplify LMP-induced by Mtb or *vice versa* **(C)**. LMP releases lysosomal enzymes, such as cathepsin B (CTSB) **(D)**. Once in the cytosol, activated CTSB supports the interaction between NLRP3 and ASC **(E)** facilitating the NLRP3-inflammasome assembly following caspase-1 recruitment **(F)**. Several molecules of ASC and caspase-1 will be recruited to NLRP3-complex leading the formation of the ASC speck core **(G)**, which amplifies IL-1 β production **(H)**. The secretion of ASC specks and IL-1 β to extracellular milieu may exacerbate inflammation **(I)**.

The results presented here identify CTSB (and possibly other cathepsins inhibited by CA074-Me) as key inducer of inflammasome assembly and activation in the context of Mtb infection (**Figure 5**). Our data also demonstrate an association of increased CTSB levels with active TB in both patients and animal models and our *in vitro* experiments with Mtb-infected macrophages indicate that these elevations may reflect increases in the intracellular levels of the mature form of the enzyme. Whether or not the latter finding is due to increased pro-enzyme synthesis was not addressed here although a previous study employing human monocyte-derived macrophages reported a decrease in cathepsin transcription following Mtb infection (79). At present, it is not clear whether this apparent discrepancy in the data reflects the host species or bone marrow versus monocyte origin of the macrophages employed in the present versus previous study. Indeed, pharmacological CTSB inhibition by dampening inflammasome-dependent IL-1β production may suppress inflammatory processes detrimental for disease outcome, such as excessive IL-1β production.

## Materials and Methods

### Mice and Rabbits

C57BL/6 mice were purchased from Taconic Farms (Hudson, NY, USA). NLRP3^−/−^, ASC^−/−^, and caspase-1^−/−^ mice were kindly provided by Dr. Karina Bortolucci from Federal University of São Paulo, Brazil. CTSB^−/−^ and CTSL^−/−^ mouse-derived materials were kindly provided by Dr. John Misasi from Harvard University, USA. All experimental procedures were in accordance with an animal study proposal approved by the NIAID Animal Care and Use Committee.

Mice and rabbits were infected as previously described (51, 80, 81). The lung samples employed were from infected animals described in previously published papers (51, 80, 81). At day 42 post-infection, different lung regions presenting with cavitation (cavity wall), granulomatous lesions, or normal lung tissue were dissected for RNA and protein analysis, and snap frozen in liquid nitrogen as previously described (51, 80).

### Human Samples

The CTSB concentration in plasma samples stored with ethyl-enediaminetetraacetic acid (EDTA) were assessed by ELISA. Samples were from 13 patients [7 males; median age, 33 years (range, 25–52 years)] with a diagnosis of active pulmonary TB confirmed by sputum culture and from 12 healthy blood donors [6 males; median age, 30 years (range, 10–58 years)] who were matched for age and sex and recruited between 2012 and 2014.

### RNA Preparation and RNA Deep Sequencing

RNA was extracted by bead beating in Trizol and column purified (Qiagen, USA) as previously described (80). Fragmentation of the whole transcriptome RNA was performed by chemical induction following the manufacture’s protocol (Applied Biosystem SOLiD Total RNA-Seq Kit; Applied Biosystems, USA). Fragmented RNA was purified and construction of the amplified whole transcriptome library performed following the manufacture’s instruction. CTSB expression was verified as previously reported (80).

### Generation of BMDMs and *In Vitro* Infection

Bone marrow-derived macrophages were generated from mouse femurs as previously described (82), with some modifications. Briefly, the femurs were flushed with 5 mL complete RPMI medium (Gibco, USA; 1 mM sodium pyruvate, 2 mM glutamine, 0.05% gentamicin, and 10% FCS) and cultivated with 30% L929 supernatant media to differentiate BMDM. An additional 10 mL of L929 cell-conditioned medium were added after 4 days of incubation. BMDMs were detached and seeded in 96-well plates at 10^5^ cells/well, containing OptiMEM media (Invitrogen, USA) at 37°C in 5% CO_2_ atmosphere.

In some experiments, we treated the cells with CA074-Me (25 µM) and Src inhibitor (10 µM) for 1 h prior to bacterial infection. BMDMs were infected with H37Rv, ρESAT-6 H37Rv, and ρRD1 H37Rv strains (a gift from Dr. Volker Briken, University of Maryland, College Park, MD, USA) at MOI of 1 or 3 for 3 h, washed and then cultivated for 5 days. Bacterial uptake was evaluated by CFU counting in BMDM treated with 0.05% saponin (Sigma-Aldrich, USA) for 10 min at different time point. Cytotoxicity induced by bacteria was analyzed in cell culture was determined by lactate dehydrogenase cytotoxicity assay kit (Caymam Chemical, USA) according to the manufacture’s protocol. In some experiments, a recombinant Mtb ESAT-6 fused with N-terminal fragment of lethal factor of *Bacillus anthracis* (LFn-ESAT6) was used to deliver ESAT-6 into the cytosol of infected and uninfected macrophages as previously described (50, 51). Anthrax-protective Ag used in this procedure was also prepared as described previously (83).

### WB and Immunoprecipitation (IP)

Samples for WB and IP were obtained from 1.5 × 10^6^ cells by lysis using NP-40 lysis buffer [150 mM NaCl, 20 mM Tris–Hcl pH 8.0, 1% Nonidet P-40 (NP-40), 10% glycerol, 2 mM EDTA]. Proteins from macrophages supernatants were concentrated by methanol/chloroform precipitation method as described previously (84). For IP, a pre-clearing step was performed in the samples by incubation with an isotype control antibody and protein A/G for 2 h at 4°C, and then washed with lysis buffer by centrifugation at 3,000 rpm for 30 s. Anti-ASC antibody (Santa Cruz, CA, USA) was added to cell lysates (1 mg/mL) and samples were continuously mixed by rotation at 4°C for 1 h, followed by Protein A/G agarose (Santa Cruz Biotechnology, USA) addition and continued overnight 4°C incubation with rotation. Beads were centrifuged at 3,000 rpm for 30 s and washed with NP-40 buffer two times prior to elution of proteins using SDS loading buffer (10% SDS, 0.6 M DTT, 30% glycerol, 0.012% bromophenol blue, at 90°C, 5 min). Prior to electrophoresis, the samples in SDS loading buffer were boiled at 95°C for 5 min. Protein transference to the nitrocellulose membrane was performed using Trans-Blot^®^ Turbo™ Transfer System Bio-Rad machine according to the instructions of the manufacturer. Nitrocellulose membranes were blocked using 1% milk diluted in 0.02% PBS-Tween20. Western blots were performed using either anti-CTSB (1:250; sc-6493), anti-caspase-1 (1:250; sc-514), anti-IL-1β (1:1,000; AF-401), anti-ASC (1:250; sc-30153), anti-βactin (1:1,000; a5316), or anti-NLRP3 (1:1,000; sc-34411).

### Cytokine Quantification and CTSB Activity

Cytokine levels in lungs and spleens homogenates were measured using commercial ELISA Kits (R&D Systems, USA) according to manufacturer’s instructions. Human total CTSB was measured in the human serum using Human Total CTSB DuoSet ELISA Kit (R&D Systems, USA) according to manufacturer’s protocol. CTSB activity in the lungs homogenates was measured using CTSB activity assay Fluorometric Kit (Abcam, USA) according to the specifications of the manufacturer. CTSB activity was visualized in Mtb-infected BMDM using a Magic Red CTSB assay Kit according to the manufacturer’s instructions (Immunochemistry, USA). Samples were examined by confocal microscopy (Nikon, Japan) using an excitation filter of 550 nm and emission filter of 620 nm.

### ASC Speck Formation

Inflammasome complex assembly was evaluated by detection of ASC speck formation using immunofluorescence microscopy. Cells were fixed with 4% paraformaldehyde buffer (pH 7.2–7.4) for 35 min, washed three times with 1× PBS and kept in ammonium chloride buffer (50 mM; pH 8.0) for 15 min, and then washed three times with 1× PBS. Fixed cells were permeabilized with 0.5% Triton-100X buffer (Sigma-Aldrich, USA) for 10 min. After permeabilization, cells were blocked with 8% milk and 10% BSA in 1× PBS to avoid nonspecific binding, followed by overnight incubation with specific primary antibody against murine ASC (1:500; Millipore) in 0.25% Tween-20 1× PBS. Cells were washed with 0.25% Tween-20 1× PBS to remove the primary antibody and incubated with anti-rabbit FITC (1:1,000; BD, USA) for 30 min. DAPI (1:1,000; Sigma-Aldrich, USA) was added to samples for nucleus staining. Images were acquired by using fluorescence microscope (Nikon, Japan).

### Statistical Analysis

Statistical analyses were performed using GraphPad Prism 4 software (GraphPad, USA). Both one-way ANOVA test and Tukey *post hoc* test were used to assess the effects of only one parameter. Differences between groups were considered significant when *p* < 0.05.

## Ethics Statement

The clinical protocol was approved by the institutional review board from the National Institute of Allergy and Infectious Diseases, National Institutes of Health (Bethesda, MD; protocol NCT01611402). Written informed consent was obtained from all study participants. The protocols, procedures, and animal care for the murine and rabbit were approved, respectively, by the Institutional Animal Care and Use Committee from the National Institutes of Health (NIH), Bethesda, MD, USA (protocol number LPD99E) and Johns Hopkins University, Baltimore, MD, USA (protocol number RB11M466). All the experiments were carried out in accordance with the recommendation of the Guide for the Care and Use of Laboratory Animals of the National Research Council of the National Academies of the United States of America (8th Edition) as mandated by the U.S. Public Health Service Policy. All animal experiments were performed in animal BSL-3 facilities maintained by the Intramural Research Program of National Institute of Allergy and Infectious Diseases (NIAID) and Johns Hopkins University.

## Author Contributions

EA, NR, MM, NM, KM-B, RP, SL, AK, and BA performed experiments. EA and BA designed experiments. MM, KM-B, WB, MD-L, AS, and BA provided materials and infrastructural support. EA, AS, and BA wrote the manuscript.

## Disclaimer

The funders had no role in study design, data collection, and interpretation or the decision to submit the work for publication.

## Conflict of Interest Statement

The authors declare that the research was conducted in the absence of any commercial or financial relationships that could be construed as a potential conflict of interest.
