# Supplementary material for: Lysosomal Cathepsin Release Is Required for NLRP3-Inflammasome Activation by Mycobacterium tuberculosis in Infected Macrophages
Source: Front Immunol. 2018 Jun 21;9:1427. doi: 10.3389/fimmu.2018.01427 (PMC6021483; doi:10.3389/fimmu.2018.01427)
Supplement: Supplementary file 1 [file data_sheet_1.PDF]

Supplementary Material

Lysosomal cathepsin release is required for NLRP3 inflammasome activation by *Mycobacterium tuberculosis* in infected macrophages

Eduardo P. Amaral, Nicolas Riteau, Mahtab Moayeri, Nolan Maier, Katrin D. Mayer-Barber, Rosana M. Pereira, Silvia L. Lage, Andre Kubler, William R. Bishai, Maria R. D'Império-Lima, Alan Sher, Bruno B. Andrade

Corresponding authors:

Bruno B. Andrade
bruno.andrade@bahia.fiocruz.br

Eduardo P. Amaral
eduardo.amaral@nih.gov

Alan Sher
asher@niaid.nih.gov

Supplementary Figures

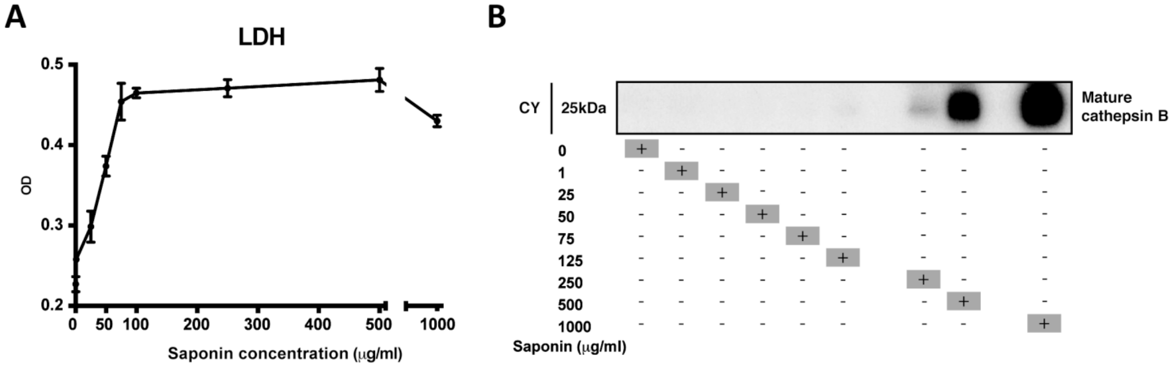

Figure S1 – Evaluation of cytosolic CSTB. To isolate CSTB into cytosol, cells were treated with different concentrations of saponin for 10 min. (**A**) LDH release was quantified in cell supernatants. (**B**) Presence of CSTB in the cytosolic fraction was examined by western-blotting. Optimal concentration of saponin to obtain cytosolic fraction was determined by the saturation of LDH release and absence of CSTB in the cytosol.

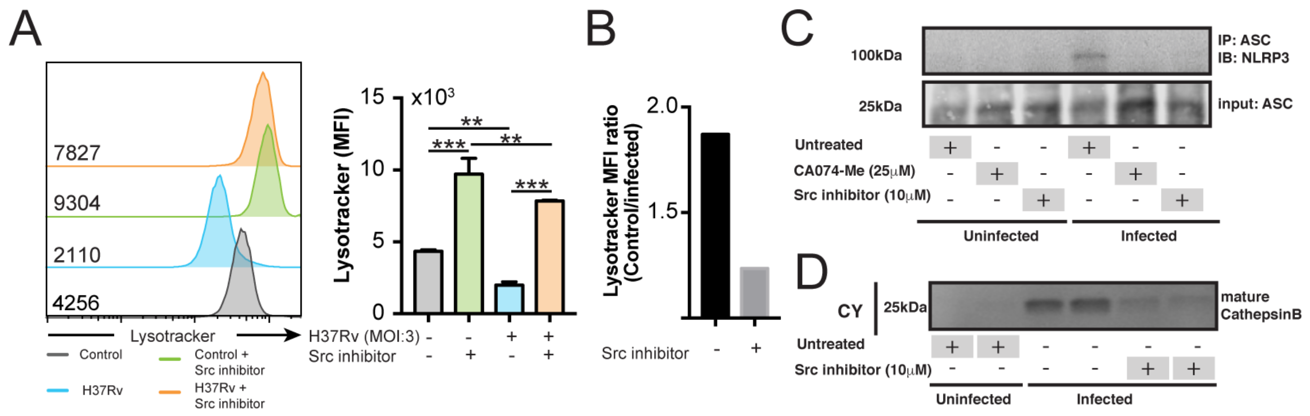

Figure S2 – Src inhibition induces lysosomal stability. Macrophages were infected with Mtb H37Rv MOI of 3 as described on Material and Methods and cells were treated or not with the Src inhibitor (10 μM) as indicated. (**A**) Cells were stained with lysotracker to verify lysosomal leakage at 24h p.i.. (**B**) Ratio of lysotracker MFI was performed to assess the difference in lysotracker staining between control and infected samples from each group as indicated. (**C**) Cell lysates were obtained after 24 h of infection. ASC was immunoprecipitated and NLRP3-coimmunoprecipitated was evaluated by NLPR3 immunoblotting. (**D**) Cytosolic fraction from BMDMs infected with Mtb H37Rv, showing the presence of mature CSTB. The data represent mean ± SEM of samples run in triplicate. Statistically significant differences are indicated (**$p < 0.01$; ***$p < 0.001$).

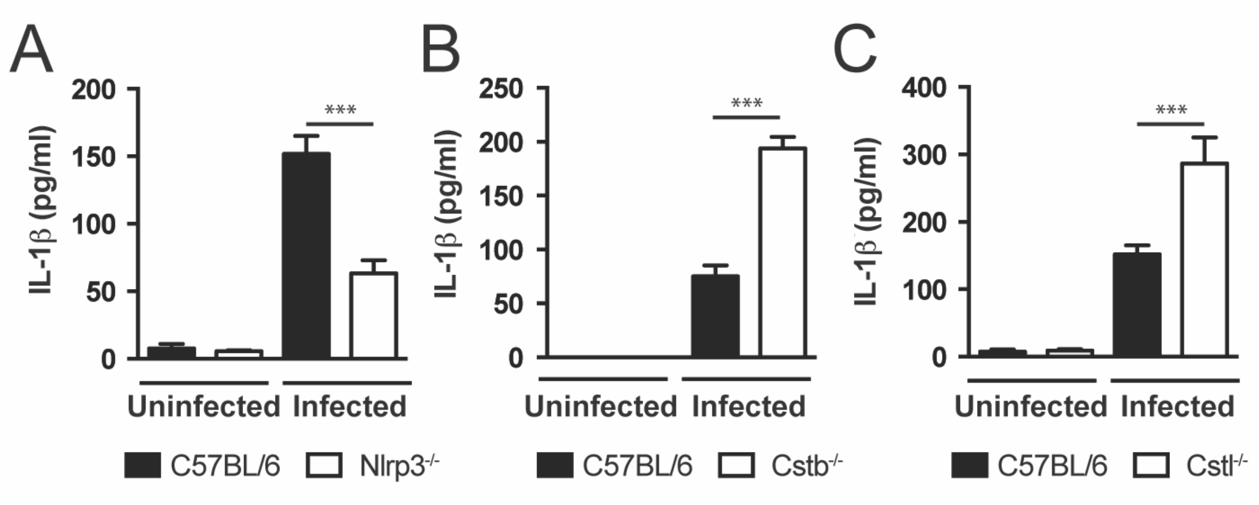

Figure S3 – Increased IL-1β production by *M. tuberculosis*-infected macrophages genetically lacking CSTB or CSTL. BMDMs were infected with H37Rv at MOI of 3 as described on Material and Methods. IL-1β level was measured in the supernatants from H37Rv-infected macrophages and uninfected cultures from Nlrp3$^{-/-}$ (**A**), Cstlb$^{-/-}$ (**B**) and Cstl$^{-/-}$ (**C**) mice and their background wild-type control. IL-1β was quantified by ELISA after 24h of infection. The data represent the means ± SEM of samples in triplicate. Statistical differences observed are shown for each indicated groups (****p* < 0.001). The data are representative of two separate experiments.

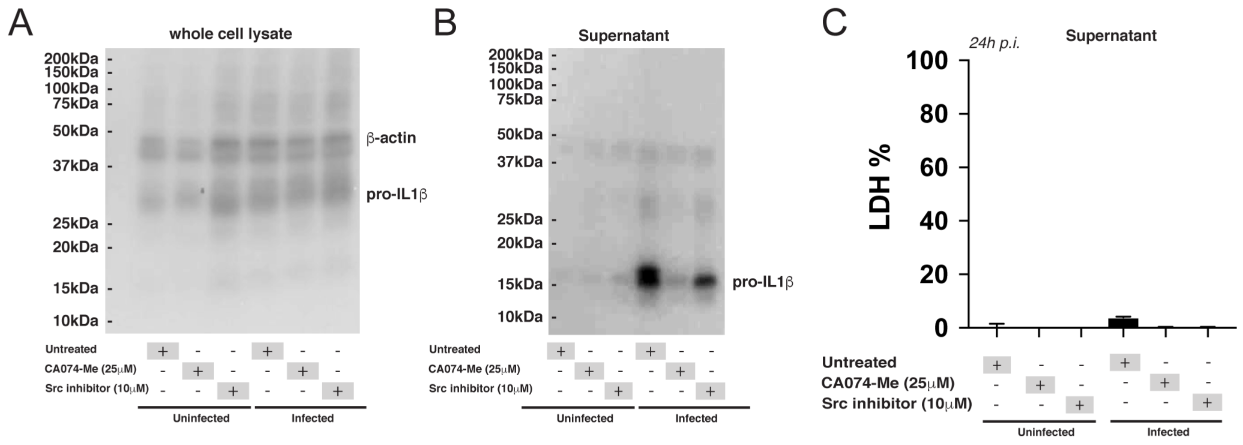

Figure S4 – IL-1β production and LDH release by *M. tuberculosis*-infected macrophages. BMDMs were infected with H37Rv at MOI of 3 as described on Material and Methods. IL-1β was measured in whole cell lysate (**A**) and in the supernatants (**B**) from H37Rv-infected macrophages and uninfected cultures after 24h of infection. (C) LDH release was measured in the supernatant from H37Rv-infected macrophages after 24h of infection. The data represent the means ± SEM of samples in triplicate. Statistical differences observed are shown for each indicated groups (****p* < 0.001). The data are representative of two separate experiments.
